# Rhamnogalacturonan from *Acmella oleracea* (L.) R.K. Jansen: Gastroprotective and Ulcer Healing Properties in Rats

**DOI:** 10.1371/journal.pone.0084762

**Published:** 2014-01-08

**Authors:** Daniele Maria-Ferreira, Luisa Mota da Silva, Daniel Augusto Gasparin Bueno Mendes, Daniela de Almeida Cabrini, Adamara Machado Nascimento, Marcello Iacomini, Thales Ricardo Cipriani, Adair Roberto Soares Santos, Maria Fernanda de Paula Werner, Cristiane Hatsuko Baggio

**Affiliations:** 1 Department of Pharmacology, Sector of Biological Sciences, Federal University of Paraná, Curitiba, PR, Brazil; 2 Department of Biochemistry and Molecular Biology, Sector of Biological Sciences, Federal University of Paraná, Curitiba, PR, Brazil; 3 Department of Physiological Sciences, Center of Biological Sciences, Federal University of Santa Catarina, Florianópolis, SC, Brazil; CIMA. University of Navarra, Spain

## Abstract

A rhamnogalacturonan (RGal) isolated from *Acmella oleracea* (L.) R.K. Jansen administered by oral route showed gastroprotective activity against acute lesions induced by ethanol. In this study, we investigated the gastric ulcer healing effect of RGal and its mechanisms of action. Intraperitoneal treatment of animals with RGal protected the gastric mucosa against acute lesions induced by ethanol, with participation of gastric mucus. Furthermore, in the chronic ulcer model, oral administration of RGal accelerates the gastric ulcer healing, accompanied by increasing of cellular proliferation and gastric mucus content, reducing inflammatory parameters and oxidative stress. In addition, the repeated 7 days-treatment of animals with RGal did not show alterations of clinical and behavioral symptoms, body and organs weights or plasmatic biochemical parameters. Collectively, these results showed that RGal has an interesting antiulcerogenic activity and could constitute an attractive molecule of interest for the development of new antiulcer agents.

## Introduction

*Acmella oleracea* (L.) R.K. Jansen (bas. *Spilanthes oleracea*; syn. *Spilanthes acmella* var. *oleracea*) is a plant of the Asteraceae family and popularly known as “jambu”, “agrião bravo” or “agrião do Pará”. In northern Brazil (Amazon region), it is commonly used as ingredient for food and in traditional medicine for the treatment of several disorders, which include toothaches, stomatitis and cold. Indeed, some biological activities have been described for *S. acmella*, such as anesthetic, anti-inflammatory, analgesic and antipyretic, antiobesity and diuretic [1]–[6]. A great number of bioactive compounds were found in *S. acmella*, such as alkylamides, phenolic compounds, coumarin and triterpenoids [7]. However, the alkylamides are the most active compounds studied in this plant, which are responsible for the anesthetic [6] and anti-inflammatory activities [5]. Recently, our group demonstrated that a polysaccharide, rhamnogalacturonan, isolated from *A. oleracea* has a gastroprotective effect against acute gastric lesions induced by ethanol [8].

It is well known that gastric ulcers develop when noxious factors overwhelm an intact mucosal defense or when the mucosal defense is impaired. The main factors that cause gastric ulcers are *Helicobacter pylori* infection and use of nonsteroidal anti-inflammatory drugs (NSAIDs) [9]. However, the structural integrity of gastric mucosa is maintained through defensive pathways such as mucus barrier, increased blood flow, inhibition of gastric acid secretion, continuous cell renewal, neutralization of reactive oxygen species (ROS), and inhibition of apoptosis [10]. Despite the availability of effective therapies, such as antagonists of H_2_ receptors and proton pump inhibitors, side effects and drug interactions has been related to long term use of these agents [11]. Therefore, new therapeutic alternatives that present a good effectiveness but fewer side effects are needed as well as therapies for the improvement of ulcer healing and the prevention of disease recurrence.

For this reason, the aim of this study was to investigate the gastric protective and healing effects of rhamnogalacturonan (RGal) isolated from *A. oleracea* in acute and chronic experimental models of gastric ulcer in rats, with the possible mechanisms underlying this activity. Besides, toxicological effect was also evaluated on 7 days-treated rats.

## Materials and Methods

### Isolation and characterization of the rhamnogalacturonan

**Figure 1:**
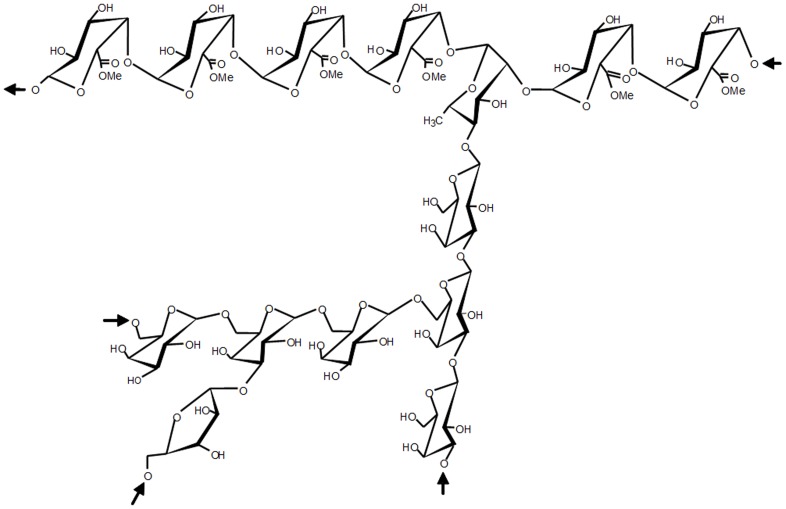
Chemical structure of rhamnogalacturonan isolated from *A. oleracea*.

The rhamnogalacturonan was isolated from leaves of *A. oleracea* (L.) R.K. Jansen as fully described in Nascimento et al.[8]. Briefly, leaves of *A. oleracea* were defatted and depigmented and then extracted with water. This aqueous extract was treated with excess EtOH to provide a crude precipitate of polysaccharides. The latter was submitted to freezing–thawing until no more precipitate appeared, and the soluble portion was treated with acetic acid, resulting in a soluble (SC) and an insoluble fraction. SC was composed of uronic acid, galactose, arabinose, rhamnose and glucose in a 15∶2∶1∶1∶0.5 molar ratio and had *M*_w_ = 226 000 g/mol [8]. Methylation analysis and nuclear magnetic resonance (NMR) spectroscopy confirmed the linkage type and main chain and side chains configuration elucidated for this polysaccharide [8], which is depicted in Fig. 1.

### Animals

Female Wistar rats (180–200 g, 2–3 months), provided by the Pontifícia Universidade Católica do Paraná colony, were maintained under standard laboratory conditions (12 h light/dark cycle, temperature of 22±2°C) with free access to standard pellet food (Nuvital®, Curitiba/PR, Brazil) and water. The animals were deprived of food (15–18 h) prior to the experiment. The experiment was performed after approval by the Institutional Animal Ethics Committee of Federal University of Paraná (CEUA/BIO-UFPR; approval number 473-B) and conducted in agreement with the ‘‘Principles of Laboratory Animal Care’’ (NIH Publication 85-23, revised 1985).

### Drugs and reagents

Alcian blue, bovine serum albumin, 1-chloro-2,4-dinitrobenzene, 2,2-diphenyl-1-picrylhydrazyl, 5,5′-dithio-bis-(2-nitrobenzoic acid), hexadecyltrimethylammonium bromide, pyrogallol, reduced glutathione, 3,3′,5,5′-tetramethylbenzidine, tris.HCl and triton X-100 were obtained from Sigma-Aldrich (St Louis, MO). All other chemicals were of analytical grade and obtained from standard commercial suppliers.

### Induction of acute gastric lesion in rats

Intragastric administration of ethanol P.A. was utilized to produce large hemorrhagic injury in the glandular stomach as described earlier by Robert et al. [12] with minor modifications. Animals were pretreated with vehicle [C: water, 1 ml/kg, per os (p.o.) or saline, 1 ml/kg, intraperitoneal (i.p.)], omeprazole (a H^+^,K^+^-ATPase inhibitor, 40 mg/kg, p.o.) or RGal (1, 3, 10 and 30 mg/kg, p.o. or 0.01, 0.1 and 1 mg/kg, i.p.), 60 min (p.o.) or 30 min (i.p.) before oral administration of ethanol P.A. (0.5 ml/200 g). After 1 h of ethanol administration, the rats were sacrificed by cervical dislocation, the stomachs were removed and the area of lesions (mm^2^) was measured using the program Image Tool 3.0.

### Determination of gastric wall mucus

Gastric wall mucus was measured using the glandular segments of the gastric mucosa with or without lesions by ethanol P.A. The gastric tissues were weighed and transferred to 0.1% Alcian Blue solution (pH 5.0), prepared in 50 mM sucrose and 0.16 mM sodium acetate solution, and stained for 2 h at room temperature. Excess dye was removed by two successive rinses with sucrose (0.25 mM) first for 15 min and after that for 45 min. Dye complexed with the gastric wall mucus was extracted with 0.5 mM magnesium chloride which was shaken for 1 min at 30 min intervals for 2 h. The resultant blue extract was then mixed with the same volume of diethyl ether and centrifuged at 1300 × *g* for 10 min. Absorbance was determined by spectrophotometry at 598 nm. Mucus amounts were quantified using standard curves of Alcian Blue (6.25–100 µg) and the results were expressed in µg of Alcian Blue/g of tissue [13].

### Induction of hypersecretion by pylorus ligature

A pylorus ligature was carefully done in rats under anesthesia according to Shay et al. [14]. Briefly, the pylorus was located and ligated with suture to maintain the gastric content into the stomach. The animals were treated with vehicle [C: water, 1 ml/kg, intraduodenal (i.d.) or p.o. or saline, 1 ml/kg, i.p.), omeprazole (40 mg/kg, p.o.) or RGal (0.1, 1 and 10 mg/kg, i.d. or 10 mg/kg, p.o. or 1 mg/kg, i.p.) immediately after (i.d. or i.p.) or 1 h before (p.o.) pylorus ligature. Four hours after pylorus ligature, the animals were sacrificed by cervical dislocation, the stomach opened and the gastric acid secretion collected. Measurements of volume and total acidity were done immediately after collection as described previously [15].

### Induction of chronic gastric ulcers by acetic acid

Chronic gastric ulcers were induced with acetic acid as described previously by Okabe et al. [16], with modifications. The rats were anaesthetized with xylazine/ketamine (7.5 mg/kg and 60 mg/kg, i.p., respectively), the abdomen was opened, the stomach exposed and 80% acetic acid (v/v, 0.5 ml) was instilled into a cylinder (6 mm of diameter) that was applied to the serosal surface of the stomach for 1 min. The acetic acid was removed by aspiration and the area of contact was washed with sterile saline. Forty eight hours after the ulcer induction, the rats were orally treated with vehicle (water, 1 ml/kg), omeprazole (40 mg/kg) or RGal (1, 3, 10 and 30 mg/kg) twice a day for 7 days.

### Assessment of gastric ulcers

On the day following the last treatment, animals were sacrificed by cervical dislocation, the stomachs removed and the ulcer area (mm^2^) was measured as length (mm) × width (mm). For histological evaluation, gastric ulcers were fixed in Alfac solution (85% alcohol 80 °GL, 10% of formaldehyde at 40% and 5% glacial acetic acid) for 16 h. After fixation, the tissue samples were dehydrated with alcohol and xylene, embedded in paraffin wax, sectioned at 5 µm and stained with hematoxylin/eosin (HE). The gastric sections were observed and photographed with a slide scanner from MetaSystems (MetaSystems MetaViewer Version 2.0.100).

### Determination of cellular proliferation

Immunohistochemical analysis of proliferating cell nuclear antigen (PCNA) was performed to determine proliferating cells in acetic acid-induced gastric ulcer. Paraffin-embedded sections were deparaffinized in xylene and hydrated throughout standard graded ethanol solutions. Sections were rinsed two times for 5 min each in PBS (pH 7.4), incubated in H_2_O_2_ solution (3% in methanol) for 10 min to inactivate endogenous peroxides. Blocking of nonspecific reaction was performed with blocking solution (1% BSA and 0.3% Triton X-100 in PBS) for 30 min. The sections were then incubated for 2 h at 4°C with goat anti-PCNA (1∶100; Santa Cruz Biotechnology Inc., CA, USA). After that, slides were rinsed in PBS (pH 7.4) and the sections were incubated in secondary antibody at room temperature for 1 h. After washing, the immunoreacted cells were then developed utilizing avidin-conjugated horseradish peroxidase (HRP) with diaminobenzidine (DAB) as substrate (BD Biosciences, San Diego, CA, USA). Finally, the specimens were counterstained with hematoxylin. PCNA-containing cells were identified by the presence of a dark reddish-brown chromogen. The gastric sections were observed and photographed with a slide scanner (MetaSystems MetaViewer Version 2.0.100) [17].

### Determination of mucin content

Mucin histochemistry was performed in accordance to Mowry and Winkler [18] and used to verify the alterations on mucin content of gastric mucus after the induction of gastric ulcer by acetic acid. Paraffin-embedded sections were deparaffinized, rehydrated, oxidized in 0.5% periodic acid for 5 min and washed in distilled water. After that, the sections were stained with Schiff's reagent for 20 min and subsequently washed with sulphurous water (three times for 2 min) and in tap water for 10 min. Finally, the slides containing sections were counterstained with hematoxylin for 20 s and dehydrated. Periodic acid-Schiff (PAS)-stained mucin-like glycoproteins positive pixels were quantified with ImageJ® software [19].

### Preparation of subcellular fractions of stomachs

Gastric ulcers were homogenized with 200 mM potassium phosphate buffer, pH 6.5 and the homogenate was used to determine the reduced glutathione (GSH) and lipid hydroperoxides (LOOH) levels and then centrifuged at 9000 × *g* for 20 min. The supernatant was used for the determination of superoxide dismutase (SOD) and glutathione *S*-transferase (GST) activities and cytokine (TNF-α, IL-1β and IL-10) levels. The pellet was used to determine the myeloperoxidase (MPO) levels.

### Protein assay

Protein concentrations of the supernatants were determined by the Bradford method (Bio-Rad, Hercules, CA, USA), using bovine serum albumin as standard and carried according to the manufacturer's instructions.

### Evaluation of inflammatory parameters

#### Determination of myeloperoxidase (MPO) levels

The levels of MPO (a marker of neutrophil infiltration) was measured according to Bradley et al. [20] and modified by De Young et al. [21]. The pellet was re-suspended in 1 ml of 80 mM potassium phosphate buffer (pH 5.4) containing 0.5% hexadecyltrimethylammonium bromide (HTAB), and it was centrifuged at 11000×*g* for 20 min at 4°C. MPO levels of supernatants were determined at 620 nm in presence of 0.017% H_2_O_2_ and 3,3′,5,5′-tetramethylbenzidine (TMB, 18.4 mM) and expressed as units of milli optic density (mO.D.)/mg of protein.

#### Determination of cytokine levels

Gastric ulcer supernatants were used to estimate the cytokine levels by enzyme-linked immunosorbent assay (ELISA). Sample aliquots of 100 µl were used to measure tumor necrosis factor alpha (TNF-α), interleukin (IL)-1β and IL-10 levels using rat cytokine ELISA kits from R&D Systems (Minneapolis, MN), according to the manufacturer's instructions. The absorbance for all cytokines studied was measured using a microplate reader at 450 and 550 nm.

### Evaluation of antioxidant system

#### Determination of reduced glutathione (GSH) levels

GSH levels in gastric mucosa were determined by the method of Sedlak and Lindsay [22]. Aliquots of tissue homogenate were mixed with 12.5% trichloroacetic acid, vortexed for 10 min and centrifuged for 15 min at 900 × *g*. Subsequently, the supernatant were mixed with TRIS buffer (0.4 M, pH 8.9) and 5,5′-dithiobis (2-nitrobenzoic acid) (DTNB, 0.01 M). Absorbance was measured by spectrophotometry at 415 nm with a microplate reader. The procedures were performed at 4°C, and the individual values were interpolated into a standard curve of GSH (0.375–3 µg) and expressed as µg/g of tissue.

#### Determination of lipid hydroperoxides (LOOH) content

The levels of LOOH were determined by the method of Ferrous Oxidation-Xylenol Orange (FOX2) as described by Jiang et al. [23]. Briefly, 10 µl of 90% methanol was added to 100 µl of homogenate, sonicated and centrifuged at 9000 × *g* for 20 minutes at 4°C. The supernatant was mixed with FOX2 reagent [4 mM butylated hydroxytoluene (BHT), 250 mM FeSO_4_, 25 mM H_2_SO_4_ and xylenol orange at 100 mM] and incubated for 30 min at room temperature. The absorbance was determined at 560 nm in a microplate reader and the concentration of LOOH was calibrated in a base of 1 mg of tissue from the homogenized sample and the results were expressed as mmol/mg of tissue.

#### Determination of gluthatione S-transferase (GST) activity

GST activity was measured using the method of Habig et al. [24]. Reactions were carried out in the presence of supernatant aliquots, 1 mM 1-chloro-2,4-dinitrobenzene (CDNB), 1 mM GSH and 100 mM potassium phosphate buffer (pH 6.5) at room temperature. Conjugation of CDNB with GSH was monitored at 340 nm for 90 s. Specific activity was calculated using an extinction coefficient of 9.6/mM/cm for GSH and the results were expressed as µmoles/min/mg of protein.

#### Determination of superoxide dismutase (SOD) activity

The method used to determine SOD activity is based in the capacity of SOD to inhibit pyrogallol autoxidation, according to Marklund and Marklund [25] and Gao et al., [26]. Pyrogallol (1 mM) was added to buffer solution (200 mM Tris HCl–EDTA, pH 8.5) and supernatant aliquots, and then vortexed for 1 min. The reaction was incubated for 20 min at room temperature, stopped with the addition of 1 N HCl and centrifuged for 4 min at 18700 × *g*. The absorbance of the resulting supernatant was measured at 405 nm using a microplate reader. The amount of SOD that inhibited the oxidation of pyrogallol by 50%, relative to the control, was defined as one unit of SOD activity. The enzymatic activity was expressed as U/mg of protein.

#### 2,2-Diphenyl-1-picrylhydrazyl (DPPH) free-radical scavenging assay

DPPH is a stable free radical that has been widely used as a tool to estimate the free-radical scavenging activity of antioxidants. The reduction capacity of the DPPH radical was determined by the decrease of absorbance induced by antioxidants, following Blois [27] and Chen et al. [28], with a few modifications. Aliquots of RGal (1, 10, 100 and 1000 µg/ml) were mixed with DPPH methanolic solution (10 µg/ml). Ascorbic acid (50 µg/ml) was used as positive control and distilled water was used as negative control. After 5 min, the absorbance was measured at 517 nm using a spectrophotometer. The individual values were interpolated into a standard curve of DPPH (0–60 µM) and expressed as μM.

### Evaluation of toxicity

During the treatment period, the body weight of all groups was recorded daily and the animals were observed for detection of clinical and behavioral signs. At the end of the treatment, the animals were sacrificed by cervical dislocation and the selected organs were removed and weighted (adrenal, heart, kidney, liver, lung, spleen, ovaries and uterus). Organ weights are reported as relative weight [(organ weight/body weight) ×100]. Serum samples were analyzed using a commercial kit (Bioclin/Quibasa, Belo Horizonte, MG, Brazil). The parameters evaluated were: alanine aminotransferase (ALT), aspartate aminotransferase (AST), creatinine and urea.

### Statistical analysis

Results were expressed as mean ± standard error of the mean (SEM) with 6–10 animals per group. Statistical significance was determined using one-way analysis of variance (ANOVA) followed by Bonferroni's test or the Kruskal-Wallis' test followed by Dunns' test using Graph-Pad software (GraphPad software, San Diego, CA, USA). IC_50_ value (concentration capable of scavenging the DPPH radicals by 50% relative to the control value) were determined by nonlinear regression analysis and reported as geometric mean. Differences were considered to be significant when p<0.05.

## Results

### Effect of RGal on acute gastric lesions induced by ethanol

As previously observed by Nascimento et al. [8], the oral treatment of animals with RGal (1, 3, 10 and 30 mg/kg) reduced the ethanol-induced gastric lesions in a dose-dependent manner with ED_50_ value of 1.5 mg/kg.

**Figure 2:**
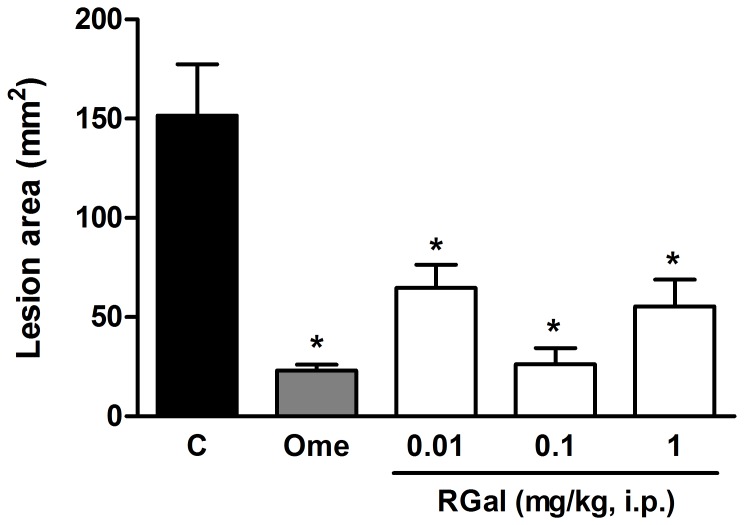
Effect of RGal on acute gastric lesions induced by ethanol P.A. in rats. The animals were treated with vehicle (C: saline, 1 ml/kg, i.p.), omeprazole (Ome: 40 mg/kg, p.o.) or RGal (0.01, 0.1 e 1 mg/kg, i.p.) 30 min before oral administration of ethanol P.A. (0.5 ml/200 g). The results are expressed as mean±S.E.M. (*n* = 8). ANOVA followed by Bonferroni's test. *P<0.05 when compared to injured group (C).

Similarly, when RGal (0.01, 0.1 and 1 mg/kg) was administered by intraperitoneal route, the gastric lesions were reduced by 57, 83 and 65%, respectively, compared to injured group [Control (C): 151.7±25.7 mm^2^]. The positive control of the test, omeprazole (40 mg/kg, p.o.), also inhibited the gastric lesions by 85% compared to injured group (Fig. 2).

### Effect of RGal on gastric wall mucus and GSH levels

The administration of ethanol P.A. decreased the gastric mucus and GSH levels by 39 and 54%, respectively, when compared to non-lesionated group (Naive: 1 251.3±163.7 µg of Alcian Blue/g of tissue and 608.5±48.0 µg/g of tissue, respectively). Oral treatment with RGal, at all doses tested, did not alter the gastric mucus and GSH levels compared to injured group (C: 758.4±39.9 µg of Alcian Blue/g of tissue and 277.2±30.6 µg/g of tissue, respectively) (Table 1). Omeprazole (40 mg/kg, p.o.) only prevented the decrease in GSH levels when compared with the injured control (Table 1).

**Table 1:** Effect of oral administration of RGal on mucus and GSH levels in acute gastric lesions induced by ethanol P.A. in rats.

Treatment	Mucus (µg of Alcian Blue/g of tissue)	GSH (µg/g of tissue)
**Naive**	1251.0±163.7	608.5±48.0
**Injured group (C: water, 1** **ml/kg)**	758.4±39.9 ^#^	277.2±30.6 ^#^
**Omeprazole (40** **mg/kg)**	583.8±99.6	550.4±71.3 *
**RGal (1** **mg/kg)**	714.4±98.9	77.4±22.4
**RGal (3** **mg/kg)**	603.9±39.7	165.0±32.4
**RGal (10** **mg/kg)**	680.4±112.6	386.0±75.7
**RGal (30** **mg/kg)**	445.4±22.2	212.0±23.0

The results were expressed as mean±S.E.M. and statistical comparison was performed using ANOVA followed by Bonferronís test. ^#^ *P*<0.05 when compared to naive. * *P*<0.05 when compared with the injured group.

Likewise, the gastric wall mucus and GSH levels were also depleted by ethanol administration when RGal was administered by the intraperitoneal route (Naive: 1 340.0±168.6 µg of Alcian Blue/g of tissue and 534.6±46.0 µg/g of tissue, respectively). Intraperitoneal administration of RGal (0.01, 0.1 and 1 mg/kg) restored the mucus levels to 1 397.7±90.2, 1 196.6±89.5 and 1 179.5±154.5 µg of Alcian Blue/g of tissue, when compared with the injured group, but did not alter the GSH levels (Table 2). In this set of experiment, omeprazole (40 mg/kg, p.o.) did not alter the gastric mucus and GSH levels compared to injured group (Table 2).

**Table 2:** Effect of intraperitoneal administration of RGal on mucus and GSH levels in acute gastric lesions induced by ethanol P.A. in rats.

Treatment	Mucus (µg of Alcian Blue/g of tissue)	GSH (µg/g of tissue)
**Naive**	1340.0±168.6	534.6±46.0
**Injured group (C: saline, 1** **ml/kg)**	724.8±51.1 ^#^	307.5±34.7 ^#^
**Omeprazole (40** **mg/kg)**	583.8±99.6	438.0±54.1
**RGal (0.01** **mg/kg)**	1398.0±90.2 *	340.2±43.5
**RGal (0.1** **mg/kg)**	1197.0±89.5 *	334.6±35.8
**RGal (1** **mg/kg)**	1180.0±154.5 *	249.3±33.8 *

The results were expressed as mean±S.E.M. and statistical comparison was performed using ANOVA followed by Bonferronís test. ^#^ *P*<0.05 when compared to naive. * *P*<0.05 when compared with the injured group.

### Effect of RGal on gastric acid secretion

Hypersecretion induced by pylorus ligature for 4 h was not changed by any tested dose of the RGal (0.1, 1, and 10 mg/kg, i.d. or 10 mg/kg, p.o. or 1 mg/kg, i.p.). Omeprazole, the positive control of the test, inhibited the gastric volume and total acidity up to 30 and 92%, respectively (Table 3).

**Table 3:** Effect of intraduodenal (i.d.), oral (p.o.) and intraperitoneal (i.p.) administration of RGal on volume and total acidity on pylorus ligature in rats.

Treatment	Volume (ml)	Total acidity (mEq[H^+^]/ml)
Control (water, 1 ml/kg, i.d)	7.12±0.87	0.078±0.010
Omeprazole (40 mg/kg, p.o.)	3.60±0.37 *	0.016±0.003 *
RGal (0.01 mg/kg, i.d.)	6.62±0.47	0.086±0.005
RGal (0.1 mg/kg, i.d.)	5.83±1.30	0.087±0.012
RGal (1 mg/kg, i.d.)	6.87±0.87	0.070±0.016
Control (water, 1 ml/kg, p.o.)	6.50±0.92	0.070±0.011
Omeprazole (40 mg/kg, p.o.)	3.12±0.31 *	0.018±0.003 *
RGal (10 mg/kg, p.o.)	9.00±1.30	0.100±0.008
Control (saline, 1 ml/kg, i.p.)	6.56±0.67	0.062±0.007
Omeprazole (40 mg/kg, p.o.)	3.60±0.37 *	0.017±0.003 *
RGal (10 mg/kg, i.p.)	6.25±0.97	0.056±0.007

The results were expressed as mean±S.E.M. and statistical comparison was performed using ANOVA followed by Bonferronís test. * P<0.05 when compared with the control group.

### Effect of RGal on chronic gastric ulcer induced by acid acetic

**Figure 3:**
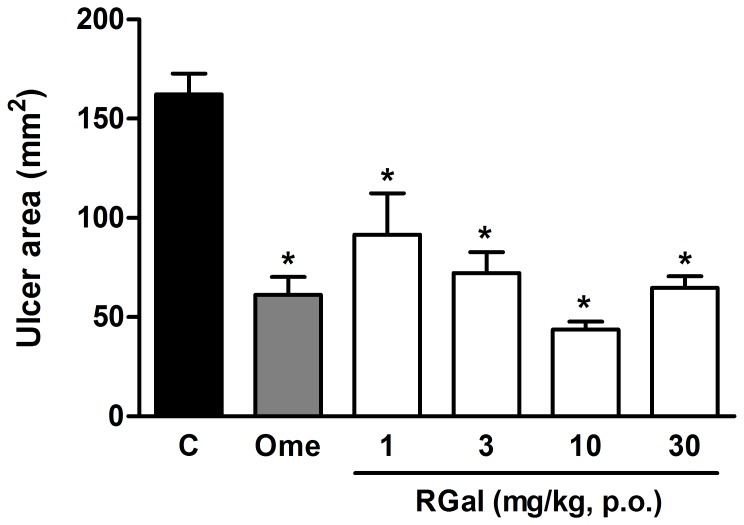
Effect of RGal on chronic gastric ulcer induced by 80% acetic acid in rats. The animals were orally treated with vehicle (C: water, 1 ml/kg), omeprazole (Ome: 40 mg/kg) or RGal (1, 3, 10 and 30 mg/kg) twice a day for seven days after the gastric ulcer induction. The results are expressed as mean±S.E.M. (*n* = 8). ANOVA followed by Bonferroni's test. *P<0.05 when compared to ulcerated group (C).

Oral administration of RGal (1, 3, 10 and 30 mg/kg, twice a day for 7 days) reduced the gastric ulcer induced by acetic acid in 43, 55, 73 and 60%, respectively. Omeprazole (40 mg/kg, p.o.) positive control of the test, also inhibited the gastric ulcer area in 62% when compared to ulcerated group [Control group (C): 162.3±10.4 mm^2^] (Fig. 3).

**Figure 4:**
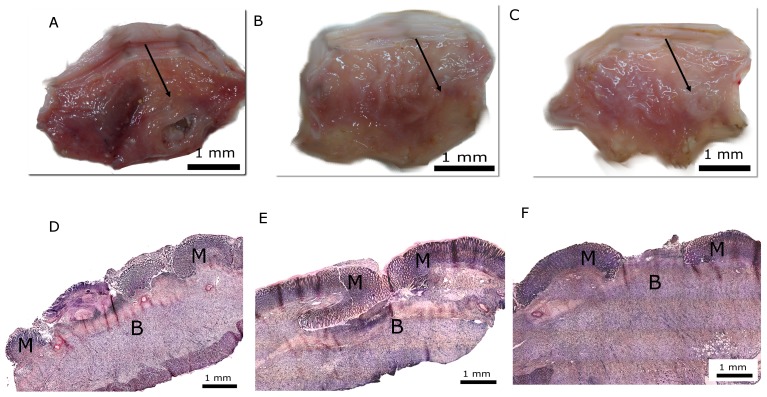
Representative macroscopic photograph of stomachs and histological hematoxylin/eosin (HE) sections (100×) of chronic gastric ulcer induced by 80% acetic acid in rats. Animals were orally treated with vehicle (water, 1 ml/kg; Panel A and D), omeprazole (40 mg/kg; Panel B and E) or RGal (10 mg/kg; Panel C and F) twice a day for seven days after the gastric ulcer induction. Bars = 1 cm (A–C) and 2 mm (D–F), where M indicates margin of ulcer and B indicates base of ulcer.

Histological analysis of the gastric ulcers showed that acetic acid promoted an extensive deep tissue injury (Fig. 4A and D). Slices from gastric ulcers treated with omeprazole (40 mg/kg, p.o.) or RGal (10 mg/kg, p.o.) demonstrated an ulcer size regression (Fig. 4B and C, respectively), arranged into columnar structures above the granulation tissue (Fig. 4E and F, respectively).

### Effect of RGal on cell proliferation

**Figure 5:**
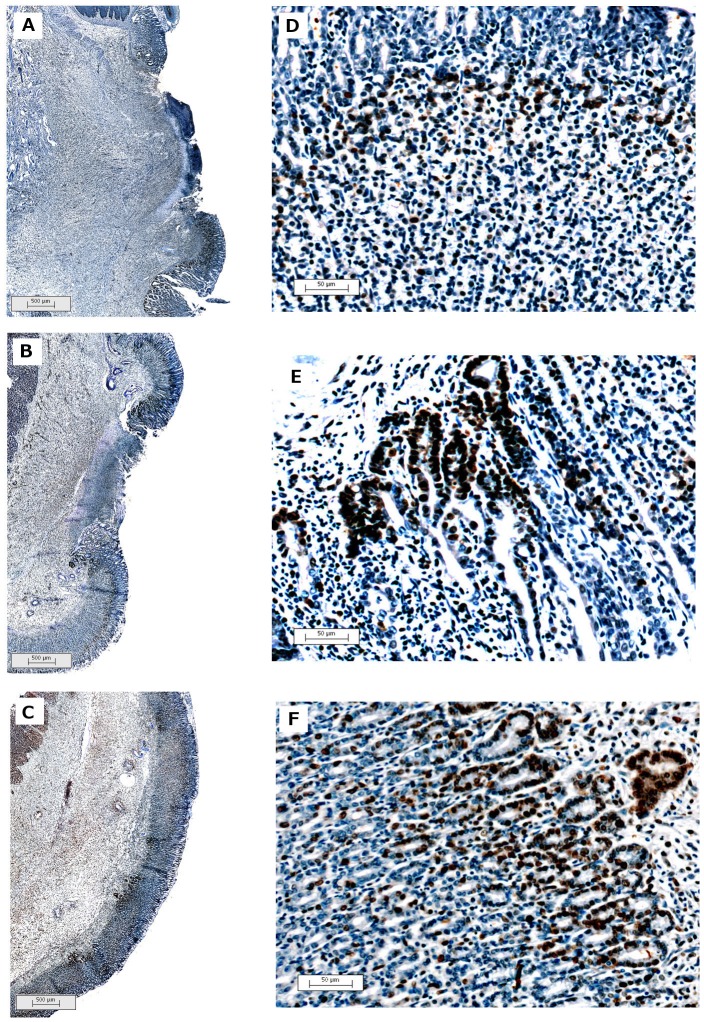
Effect of RGal on the immunohistochemical staining for PCNA in chronic gastric ulcer induced by 80% acetic acid in rats. Photomicrographs represent PCNA immunoreactivity of groups orally treated with vehicle (water, 1 ml/kg; Panels A and D), omeprazole (40 mg/kg; Panels B and E) or RGal (10 mg/kg; Panels C and F) twice a day for seven days after the gastric ulcer induction. Panels A-C: magnification = 7×, bars = 500 µm; Panels D-F: magnification = 100×, bars = 50 µm.

According to histological analysis, the oral treatment of animals with omeprazole (40 mg/kg) or RGal (10 mg/kg) showed a significant increasing of PCNA immunoreactivity in ulcerated gastric mucosa by 121 and 155%, respectively (Fig. 5B and E and Fig. 5C and F, respectively), which is characterized by brown color and indicates the proliferating cells, when compared to ulcerated group (C: 129.3±31.4) (Fig. 5A and D, Table 4).

**Table 4:** Effect of oral administration of RGal on the immunohistochemical staining for PCNA and on the histochemical staining for mucin (PAS) in chronic gastric ulcer induced by 80% acetic acid in rats.

Treatment	PCNA (number of proliferating cells)	PAS stained mucin (pixels/field × 10^4^)
**Ulcerated group (C: water, 1 ml/kg)**	129.3±31.4	10.55±0.19
**Omeprazole (40** **mg/kg)**	285.5±28.2 *	17.47±0.24 *
**RGal (10** **mg/kg)**	329.2±49.3 *	16.94±0.60 *

The results were expressed as mean±S.E.M. and statistical comparison was performed using ANOVA followed by Bonferronís test. * *P*<0.05 when compared with the ulcerated group.

### Effect of RGal on gastric mucin content

**Figure 6:**
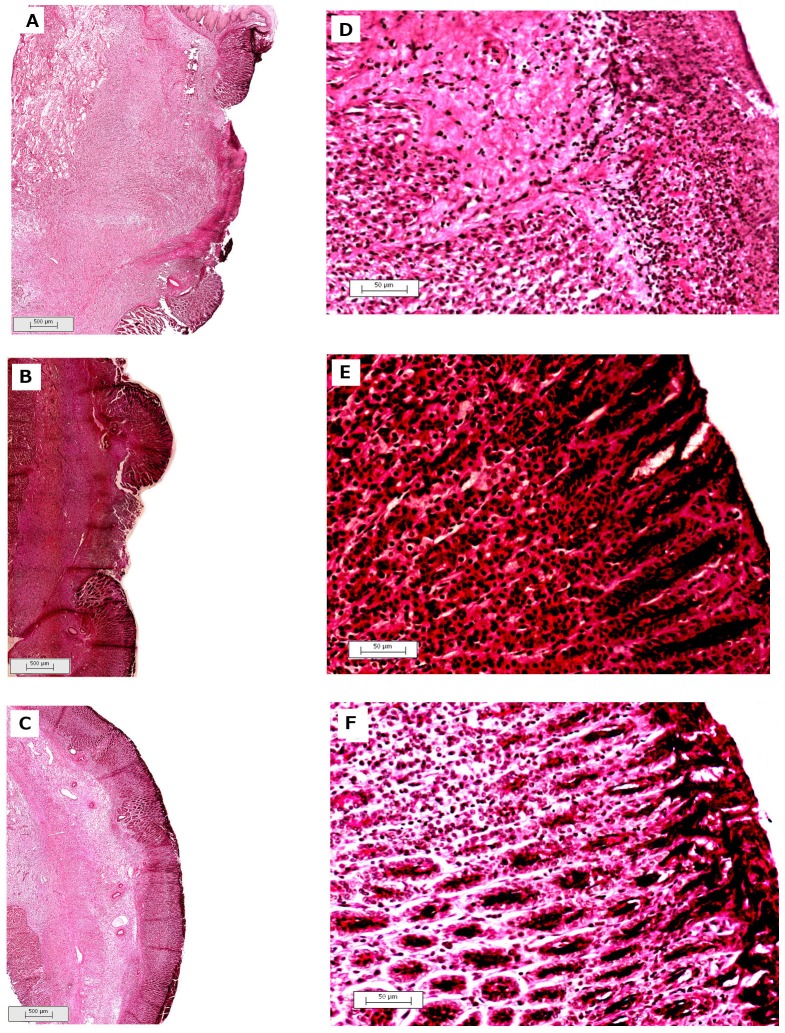
Effect of RGal on histochemical staining for mucin-like glycoproteins (PAS) in chronic gastric ulcer induced by 80% acetic acid in rats. Representative images of groups orally treated with vehicle (water, 1 ml/kg; Panels A and D), omeprazole (40 mg/kg; Panels B and E) or RGal (10 mg/kg; Panels C and F) twice a day for seven days after the gastric ulcer induction. Panels A-C: magnification = 7×, bars = 500 µm; Panels D-F: magnification = 100×, bars = 50 µm.

The results depicted in Fig. 6A and D show that acetic acid application on gastric mucosa decreased the mucin-like glycoproteins. However, oral administration of omeprazole (40 mg/kg) or RGal (10 mg/kg) increased PAS-staining for mucin in 65 and 60%, respectively (Fig. 6B and E and Fig. 6C and F, respectively), when compared to the ulcerated group (C: 10.55±0.19 pixels/field × 10^4^) (Table 4).

### Effect of RGal on inflammatory parameters

**Figure 7:**
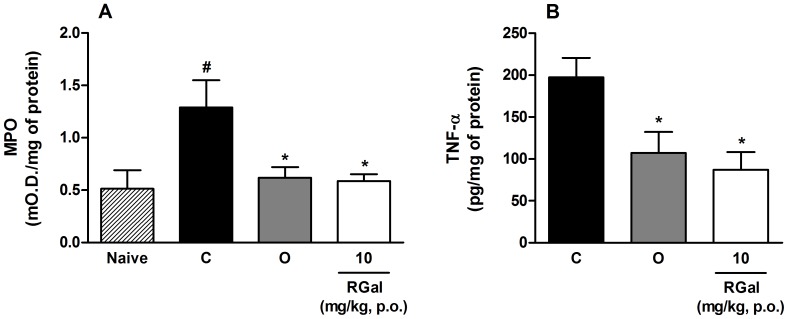
Effects of RGal on MPO (Panel A) and TNF-α (Panel B) levels in chronic gastric ulcer induced by 80% acetic acid in rats. The animals were orally treated with vehicle (C: water, 1 ml/kg), omeprazole (Ome: 40 mg/kg) or RGal (10 mg/kg) twice a day for seven days after the gastric ulcer induction. Naive (N): non-ulcerated group. The results are expressed as mean±S.E.M. (*n* = 8). ANOVA followed by Bonferroni's test. ^#^ *P*<0.05 when compared to naive (N). * *P*<0.05 when compared to ulcerated group (C).

The values of MPO levels were increased by 152% in ulcerated gastric mucosa when compared to non-ulcerated stomachs (Naive: 0.51±0.18 mO.D./mg of protein) (Fig. 7A). Oral treatment of rats with RGal (10 mg/kg) and omeprazole (40 mg/kg) significantly reduced MPO levels in 54 and 52%, respectively, when compared to ulcerated group (C: 1.29±0.26 mO.D./mg of protein) (Fig. 7A).

The results presented in Fig. 7B show that RGal (10 mg/kg, p.o.) decreased the TNF-α levels by 56% compared to ulcerated group (C: 197.5±23.0 pg/mg of protein), while IL-1β and IL-10 levels were unchanged (Table 5).

**Table 5:** Effects of oral administration of RGal in IL-1β and IL-10 levels in chronic gastric ulcer induced by 80% acetic acid in rats.

Treatment	IL-1β (pg/mg of protein)	IL-10 (pg/mg of protein)
**Ulcerated group (C: water, 1** **ml/kg)**	356.5±66.2	141.1±20.2
**Omeprazole (40** **mg/kg)**	396.3±41.3	103.8±12.5
**RGal (10** **mg/kg)**	366.5±56.3	92.8±11.5

The results were expressed as mean±S.E.M. and statistical comparison was performed using ANOVA followed by Bonferronís test. * P<0.05 when compared with the ulcerated group.

### Effect of RGal on antioxidant system

The ulcer induction by acetic acid decreased the GSH levels and increased the LOOH content in 57% and 50%, respectively, compared to non-ulcerated group (Naive: 658.2±62.3 µg/g of tissue and 46.0±5.7 mmol/mg of tissue (Table 6). Oral treatment with RGal at 10 mg/kg partially prevented the depletion of GSH levels and restored the LOOH content to 381.1±20.8 µg/g of tissue and 34.3±6.4 mmol/mg of tissue, respectively, when compared to the ulcerated group (Table 6). Omeprazole (40 mg/kg, p.o.) also partially prevented the depletion of GSH levels when compared to ulcerated group (Table 6).

**Table 6:** Effect of oral administration of RGal on GSH and LOOH levels and SOD and GST activity in chronic gastric ulcer induced by 80% acetic acid in rats.

Treatment	GSH (µg/g of tissue)	LOOH (mmol/mg of tissue)	SOD (U/mg of protein)	GST (nmol/min/mg of protein)
Naive	658.2±62.3	46.0±5.7	13.77±1.02	301.2±15.4
Ulcerated group (C: water, 1 ml/kg)	225.1±44.2 ^#^	69.0±7.1 ^#^	9.89±0.35 ^#^	222.8±14.5 ^#^
Omeprazole (40 mg/kg)	359.2±23.0 *	55.3±7.8	13.15±1.01	289.7±16.5 *
RGal (10 mg/kg)	381.1±20.8 *	34.3±6.4 *	13.50±0.87 *	312.9±13.6 *

The results are expressed as means±S.E.M. and statistical comparison was performed ANOVA followed by Bonferroni's test. ^#^ *P*<0.05 when compared to naive and * *P*<0.05 when compared to ulcerated group.

In acetic acid-induced gastric ulcers, the SOD and GST activities were decreased in 28 and 26% compared to the non-ulcerated group (Table 6). However, the oral administration of RGal (10 mg/kg) restored the SOD and GST activities to 13.50±0.87 U/mg of protein and 312.9±13.6 µmoles/min/mg of protein when compared to ulcerated group (C: 9.89±0.35 U/mg of protein and 222.8±14.5 µmoles/min/mg of protein) (Table 6). Omeprazole (40 mg/kg, p.o.), the positive control, did not alter the SOD activity but restored the GST activity (Table 6).

**Figure 8:**
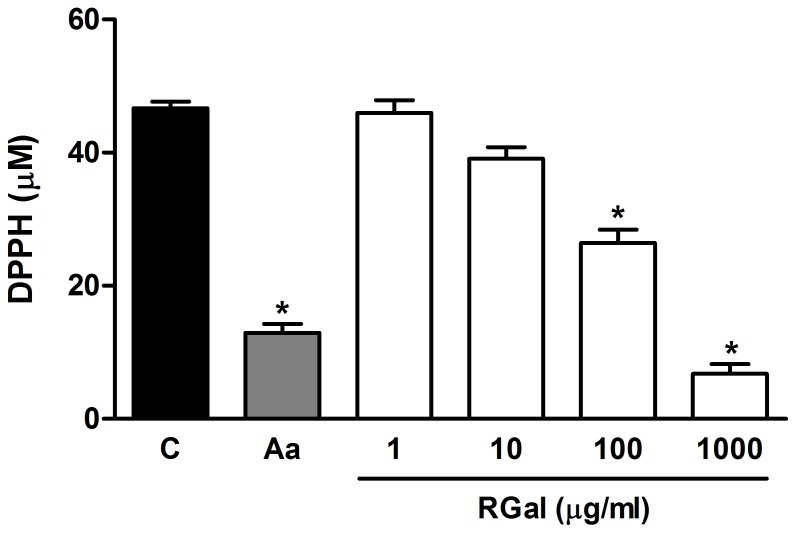
Effect of RGal on the ability to scavenge the free-radical DPPH *in vitro*. The figure shows the scavenging of DPPH radical by RGal (1, 10, 100 and 1000 µg/ml) or ascorbic acid (AA, 50 µg/ml) *in vitro*. The results are expressed as mean±S.E.M. (*n* = 8). ANOVA followed by Bonferroni's test. * *P*<0.05 when compared to control group (C).

In the *in vitro* DPPH assay, RGal scavenged the DPPH radicals in a concentration-dependent manner with IC_50_ value of 126.9 µg/ml (Fig. 8). Ascorbic acid, used as a positive control, also reduced the DPPH levels by 72% compared to control group (C: 46.7±1.0 µg/ml) (Fig. 8).

### Effects of RGal on body and organ weights

The animals treated with RGal (10 mg/kg), for 7 days twice a day by oral route, did not produce any visible signs of toxicity or alterations in body and organ weights when compared to control animals (data not shown). Besides, no changes were observed on biochemical parameters analyzed: AST, ALT, creatinine or urea (data not shown).

## Discussion

Gastric ulcer is considered a global health problem with consequential high morbidity and considerable mortality rates [29]. Considering the growing concern that has been raised about the adverse effects of classic treatment (PPIs and H_2_ antagonists) against gastric ulcers, new equally effective drugs are interesting and needed. In light of these considerations, polysaccharides recently became the focus of studies for the treatment of several disturbs. Recent works demonstrated that polysaccharides isolated from different sources exhibited important pharmacological activities, as antitumoral, immunomodulatory, antinociceptive and gastroprotective [30]–[33]. Interestingly, our group showed that the polysaccharide rhamnogalacturonan (RGal) isolated from *A. oleracea* protected the gastric mucosa against acute lesions induced by ethanol in rats, with an ED_50_ of 1.5 mg/kg when administered by oral route.

Since ethanol is responsible for depletion of defensive mechanisms such as mucus barrier and non-proteic sulphydrilic groups (mainly GSH) [34]–[35], additional experiments demonstrated that this gastroprotection promoted by oral administration of RGal is not related to these protective factors of gastric mucosa. However, when the animals were treated with RGal by intraperitoneal route, the inhibition of ethanol-induced gastric lesions seems to be attributed to maintenance of the mucus levels besides to discard the formation of a physical barrier on the gastric mucosa. Continuing with the evaluation of the defensive pathways, our results also ruled out the inhibition of gastric acid secretion as mechanism of gastroprotection of RGal. Three different routes (i.d., p.o. or i.p.) of RGal administration in animals with gastric hypersecretion induced by pylorus ligature did not alter both volume and total acidity of gastric content. These results are consistent with data obtained with an arabinogalactan isolated from *Maytenus ilicifolia*, which also did not reduce the gastric acid secretion of rats [36]. On the contrary, Yamada [31] showed that a pectic polysaccharide possess anti-secretory activity, suggesting that the structural type of polysaccharides could influence their activity.

Additionally, the acetic acid ulcer model, established by Takagi et al. [37], was the one that reflects human peptic ulcer disease from the view of macroscopic and microscopic observation [38]. For this reason, this model has been widely used to study the mechanism of ulcer healing and to evaluate the anti-ulcer effect of several compounds. Interestingly, RGal showed a gastric healing property with participation of cellular proliferation, gastric mucin, antioxidant system and inhibition of inflammatory parameters.

Ulcer healing is a complex process of tissue regeneration, which involves cell migration, proliferation, re-epithelization, gland reconstruction, angiogenesis and matrix deposition, leading to scar formation [for review see Tarnawski and Ahluwalia [9]]. Our results showed that RGal treatment accelerated the healing of chronic gastric ulcer in rats, confirmed by histological analysis where it was observed the contraction of the ulcer base. Similarly, some studies also found gastric ulcer healing effects of polysaccharides isolated from medicinal plants and mushroom [39]–[41]. According with these findings, RGal increased the number of PCNA-positive cells; a nuclear polypeptide (PCNA) which serves as tissue marker of cell proliferation [42]. This data suggests that the restoration of epithelial continuity promoted by RGal occurs through epithelial cell proliferation. Furthermore, the gastric wall mucus significantly contributes to a better re-epithelization and, consequently, to the repair of gastric mucosal damage [38]. As mentioned before, gastric wall mucus is an important protective factor of gastric mucosa and it is the first line of defense against acid secretion due to formation of a viscous, elastic, adherent and transparent gel, characteristic promoted by its composition [95% water and 5% glycoproteins (mucins)] [35], [43]. As expected, the treatment of animals with RGal was able to prevent the reduction of mucin-like glycoproteins stained with PAS, which indicates the involvement of gastric mucus on the healing activity of this compound. Corroborating our observations, Srikanta et al. [40] also reported an enhanced mucin production by a polysaccharide during gastric ulcer healing.

It is well known that the ulcers result from the tissue necrosis triggered by mucosal ischemia, free radical formation and cessation of oxygen and nutrient delivery [9]. All these events initiate an inflammatory phase of ulcer development with recruitment and metabolic activation of neutrophils and macrophages, releasing pro-inflammatory cytokines (TNF-α and IL-1β) and generating free radicals [9], [44]. The inhibition of MPO (a marker of neutrophils infiltration) and TNF-α levels by RGal treatment suggests a reduction of neutrophils migration at the ulcer site and consequently promotes an improvement of the gastric inflammatory process induced by acetic acid. Furthermore, this may contribute to the reduction of neutrophil-dependent ROS formation. In fact, the oxidative damage of the mucosa by reactive oxygen species are among the major factors involved in gastric ulceration [40]. Accordingly, we found an increase in the ROS generation in ulcerated gastric mucosa as evidenced by increased LOOH levels, depletion of GSH content and reduced SOD and GST activity. However, oral administration of RGal exhibited antioxidant mechanisms restoring the LOOH and GSH contents and SOD and GST activities to basal levels, which may accelerate the ulcer healing process by scavenging of free radicals. These results are consistent with the DPPH free-radical scavenging property presented by this polysaccharide. Besides, in agreement with our data, another polysaccharide (arabinogalactan) with gastroprotective effect also showed DPPH scavenging activity [36]. Indeed, the inhibition of neutrophils and the scavenging of hydroxyl radicals enhance the quality of gastric ulcer healing in rats [45]–[46]. It is important to note that RGal presents these activities indicating a likely cicatrization of ulcer with quality and hence ulcer recurrence.

Also of interest are the results concerning the subchronic exposition to RGal, where the animals did not exhibit clinical and behavioural signs, alterations in body and organs weights or in some biochemical parameters, evidencing the possible safety of this polysaccharide but further studies are necessary to fully exclude the toxicity of RGal.

In conclusion, RGal isolated from *Acmella oleracea* presented gastric protective and healing properties in rats. These effects could be associated to increasing cellular proliferation, maintenance of gastric mucus, reduction of inflammatory process and modulation of antioxidant mechanisms. However, additional studies are required to investigate complementary mechanisms involved in the effects produced by RGal.

## References

[1] Chakraborty A, Devi BR, Sanjebam R, Khumbong S, Thokchom IS (2010) Preliminary studies on local anesthetic and antipyretic activities of *Spilanthes acmella* Murr. in experimental animal models. Indian J Pharmacol 42: 277–279. 21206617 10.4103/0253-7613.70106 PMC2959208

[2] Ratnasooriya WD, Pieris KP, Samaratunga U, Jayakody JR (2004) Diuretic activity of *Spilanthes acmella* flowers in rats. J Ethnopharmacol 91: 317–320. 15120455 10.1016/j.jep.2004.01.006

[3] Ekanem AP, Wang M, Simon JE, Moreno DA (2007) Antiobesity properties of two African plants (*Afromomum meleguetta* and *Spilanthes acmella*) by pancreatic lipase inhibition. Phytother Res 21: 1253–1255. 17705140 10.1002/ptr.2239

[4] Chakraborty A, Devi RK, Rita S, Sharatchandra K, Singh TI (2004) Preliminary studies on antiinflammatory and analgesic activities of *Spilanthes acmella* in experimental animal models. Indian J Pharmacol 36: 148–150. 10.4103/0253-7613.70106 PMC2959208 21206617

[5] Wu LC, Fan NC, Lin MH, Chu IR, Huang SJ, et al (2008) Anti-inflammatory effect of spilanthol from *Spilanthes acmella* on murine macrophage by down-regulating LPS-induced inflammatory mediators. J Agric Food Chem 56: 2341–2349. 18321049 10.1021/jf073057e

[6] Ley JP, Krammer G, Looft J, Reinders G, Bertram H (2006) Structure-activity relationships of trigeminal affects for artificial an naturally occurring alkamides related to spilanthol. Dev Food Sci 43: 21–24.

[7] Prachayasittikul S, Suphapong S, Worachartcheewan A, Lawung R, Ruchirawat S, et al (2009) Bioactive metabolites from *Spilanthes acmella* Murr. Molecules 14: 850–867. 19255544 10.3390/molecules14020850 PMC6253828

[8] Nascimento AM, de Souza LM, Baggio CH, Werner MF, Maria-Ferreira D, et al (2013) Gastroprotective effect and structure of a rhamnogalacturonan from *Acmella oleracea* . Phytochemistry 85: 137–142. 23014505 10.1016/j.phytochem.2012.08.024

[9] Tarnawski AS, Ahluwalia A (2012) Molecular mechanisms of epithelial regeneration and neovascularization during healing of gastric and esophageal ulcers. Curr Med Chem 19: 16–27. 22300072 10.2174/092986712803414088

[10] Palileo C, Kaunitz JD (2011) Gastrointestinal defense mechanisms. Curr Opin Gastroenterol 27: 543–548. 21897225 10.1097/MOG.0b013e32834b3fcb

[11] DeVault KR, Talley NJ (2009) Insights into the future of gastric acid suppression. Nat Rev Gastroenterol Hepatol 6: 524–532. 19713987 10.1038/nrgastro.2009.125

[12] Robert A, Nezamis JE, Lancaster C, Hanchar AJ (1979) Cytoprotection by prostaglandins in rats. Prevention of gastric necrosis produced by alcohol, HCl, NaOH, hypertonic NaCl, and thermal injury. Gastroenterology 77: 433–443. 456839

[13] Corne SJ, Morrissey SM, Woods RJ (1974) Proceedings: A method for the quantitative estimation of gastric barrier mucus. J Physiol 242: 116P–117P. 4142046

[14] Shay H, Komarov SA, Fels SS, Meranze D, Gruenstein M, et al (1945) A simple method for the uniform production of gastric ulceration in the rat. Gastroenterology 5: 43–61.

[15] Baggio CH, De Martini Otofuji G, de Souza WM, de Moraes Santos CA, Torres LM, et al (2005) Gastroprotective mechanisms of indole alkaloids from *Himatanthus lancifolius* . Planta Med 71: 733–738. 16142637 10.1055/s-2005-871286

[16] Okabe S, Roth JL, Pfeiffer CJ (1971) A method for experimental, penetrating gastric and duodenal ulcers in rats. Observations on normal healing. Am J Dig Dis 16: 277–284. 5554507 10.1007/BF02235252

[17] Potrich FB, Allemand A, da Silva LM, Dos Santos AC, Baggio CH, et al (2010) Antiulcerogenic activity of hydroalcoholic extract of *Achillea millefolium* L.: involvement of the antioxidant system. J Ethnopharmacol 130: 85–92. 20420892 10.1016/j.jep.2010.04.014

[18] Mowry R, Winkler CH (1956) The coloration of acidic carbohydrates of bacteria and fungi in tissue sections with special reference to capsules of *Cryptococcus neoformans*, Pneumococci and Staphilococci. Am J Pathol 32: 628–629.

[19] Pereira IT, Burci LM, da Silva LM, Baggio CH, Heller M, et al (2012) Antiulcer effect of bark extract of *Tabebuia avellanedae*: activation of cell proliferation in gastric mucosa during the healing process. Phytother Res 27: 1067–1073. 22969019 10.1002/ptr.4835

[20] Bradley PP, Priebat DA, Christensen RD, Rothstein G (1982) Measurement of cutaneous inflammation: estimation of neutrophil content with an enzyme marker. J Invest Dermatol 78: 206–209. 6276474 10.1111/1523-1747.ep12506462

[21] De Young LM, Kheifets JB, Ballaron SJ, Young JM (1989) Edema and cell infiltration in the phorbol ester-treated mouse ear are temporally separate and can be differentially modulated by pharmacologic agents. Agents Actions 26: 335–341. 2567568 10.1007/BF01967298

[22] Sedlak J, Lindsay RH (1968) Estimation of total, protein-bound, and nonprotein sulfhydryl groups in tissue with Ellman's reagent. Anal Biochem 25: 192–205. 4973948 10.1016/0003-2697(68)90092-4

[23] Jiang ZY, Hunt JV, Wolff SP (1992) Ferrous ion oxidation in the presence of xylenol orange for detection of lipid hydroperoxide in low density lipoprotein. Anal Biochem 202: 384–389. 1519766 10.1016/0003-2697(92)90122-n

[24] Habig WH, Pabst MJ, Jakoby WB (1974) Glutathione S-transferases. The first enzymatic step in mercapturic acid formation. J Biol Chem 249: 7130–7139. 4436300

[25] Marklund S, Marklund G (1974) Involvement of the superoxide anion radical in the autoxidation of pyrogallol and a convenient assay for superoxide dismutase. Eur J Biochem 47: 469–474. 4215654 10.1111/j.1432-1033.1974.tb03714.x

[26] Gao R, Yuan Z, Zhao Z, Gao X (1998) Mechanism of pyrogallol autoxidation and determination of superoxide dimutase enzyme activity. Bioelectrochem Bioenerg 45: 41–45.

[27] Blois MS (1958) Antioxidant determinations by use of a stable free radical. Nature 181: 1199–1200.

[28] Chen FA, Wu AB, Chen CY (2004) The influence of treatments on the free radical scavenging activity of burdock and variations of its activity. Food Chem 86: 479–484.

[29] Malfertheiner P, Chan FK, McColl KE (2009) Peptic ulcer disease. Lancet 374: 1449–1461. 19683340 10.1016/S0140-6736(09)60938-7

[30] Srivastava R, Kulshreshtha DK (1989) Bioactive polysaccharides from plants. Phytochemistry 28: 2877–2883.

[31] Yamada H (1994) Pectic polysaccharides from chinese herbs - structure and biological-activity. Carbohydrate Polymers 25: 269–276.

[32] Nergard CS, Diallo D, Inngjerdingen K, Michaelsen TE, Matsumoto T, et al (2005) Medicinal use of *Cochlospermum tinctorium* in Mali Anti-ulcer-, radical scavenging- and immunomodulating activities of polymers in the aqueous extract of the roots. J Ethnopharmacol 96: 255–269. 15588678 10.1016/j.jep.2004.09.018

[33] Baggio CH, Freitas CS, Marcon R, Werner MF, Rae GA, et al (2012) Antinociception of beta-D-glucan from *Pleurotus pulmonarius* is possibly related to protein kinase C inhibition. Int J Biol Macromol 50: 872–877. 22085751 10.1016/j.ijbiomac.2011.10.023

[34] Hirschowitz BI (1983) Lessons from the U.S. multicenter trial of ranitidine treatment for duodenal ulcer. Journal of Clinical Gastroenterology 5 Suppl 1115–122. 6317737 10.1097/00004836-198312001-00011

[35] Repetto MG, Llesuy SF (2002) Antioxidant properties of natural compounds used in popular medicine for gastric ulcers. Braz J Med Biol Res 35: 523–534. 12011936 10.1590/s0100-879x2002000500003

[36] Baggio CH, Freitas CS, Twardowschy A, dos Santosa AC, Mayer B, et al (2012) In vivo/in vitro studies of the effects of the type II arabinogalactan isolated from *Maytenus ilicifolia* Mart. ex Reissek on the gastrointestinal tract of rats. Z Naturforsch C 67: 405–410. 23016280 10.1515/znc-2012-7-808

[37] Takagi K, Okabe S, Saziki R (1969) A new method for the production of chronic gastric ulcer in rats and the effect of several drugs on its healing. Jpn J Pharmacol 19: 418–426. 5307474 10.1254/jjp.19.418

[38] Okabe S, Amagase K, Takeuchi K (2012) Acetic acid ulcer model – state of the art in 2012. In: Filaretova LP, Takeuchi K, editors. Cell/Tissue Injury and Cytoprotection/Organoprotection in the Gastrointestinal Tract: Mechanisms, Prevention and Treatment. Basel: Karger. pp. 32–40.

[39] Ye YN, So HL, Liu ES, Shin VY, Cho CH (2003) Effect of polysaccharides from *Angelica sinensis* on gastric ulcer healing. Life Sci 72: 925–932. 12493573 10.1016/s0024-3205(02)02332-9

[40] Srikanta BM, Sathisha UV, Dharmesh SM (2010) Alterations of matrix metalloproteinases, gastric mucin and prostaglandin E(2) levels by pectic polysaccharide of swallow root (*Decalepis hamiltonii*) during ulcer healing. Biochimie 92: 194–203. 19853004 10.1016/j.biochi.2009.10.005

[41] Gao Y, Tang W, Gao H, Chan E, Lan J, et al (2004) *Ganoderma lucidum* polysaccharide fractions accelerate healing of acetic acid-induced ulcers in rats. J Med Food 7: 417–421. 15671683 10.1089/jmf.2004.7.417

[42] Czyzewska J, Guzinska-Ustymowicz K, Pryczynicz A, Kemona A, Bandurski R (2009) Immunohistochemical evaluation of Ki-67, PCNA and MCM2 proteins proliferation index (PI) in advanced gastric cancer. Folia Histochem Cytobiol 47: 289–296. 19995716 10.2478/v10042-009-0042-y

[43] Phillipson M, Johansson ME, Henriksnas J, Petersson J, Gendler SJ, et al (2008) The gastric mucus layers: constituents and regulation of accumulation. Am J Physiol Gastrointest Liver Physiol 295: G806–812. 18719000 10.1152/ajpgi.90252.2008

[44] Fialkow L, Wang Y, Downey GP (2007) Reactive oxygen and nitrogen species as signaling molecules regulating neutrophil function. Free Radic Biol Med 42: 153–164. 17189821 10.1016/j.freeradbiomed.2006.09.030

[45] Arakawa T, Watanabe T, Tanigawa T, Tominaga K, Fujiwara Y, et al (2012) Quality of ulcer healing in gastrointestinal tract: its pathophysiology and clinical relevance. World J Gastroenterol 18: 4811–4822. 23002355 10.3748/wjg.v18.i35.4811 PMC3447265

[46] Naito Y, Yoshikawa T, Matsuyama K, Yagi N, Arai M, et al (1995) Effects of oxygen radical scavengers on the quality of gastric ulcer healing in rats. J Clin Gastroenterol 21 Suppl 1S82–86. 8774996

